# Enhancement of Chemokine mRNA Expression by Toll-Like Receptor 2 Stimulation in Human Peripheral Blood Mononuclear Cells of Patients with Atopic Dermatitis

**DOI:** 10.1155/2020/1497175

**Published:** 2020-03-19

**Authors:** Yangyang Yu, Dongxu Lin, Xiaoqiong Cai, Danni Cui, Ran Fang, Wei Zhang, Bo Yu, Xiaomei Wang

**Affiliations:** ^1^Shenzhen University Health Science Center, Shenzhen, Guangdong, China; ^2^Institute of Urology and Department of Urology, Tongji Hospital, Tongji Medical College, Huazhong University of Science and Technology, Wuhan, Hubei, China; ^3^Shenzhen Key Laboratory for Translational Medicine of Dermatology, Biomedical Research Institute, Shenzhen Peking University-The Hong Kong University of Science and Technology Medical Center, Shenzhen, Guangdong, China; ^4^Department of Dermatology, Peking University Shenzhen Hospital, Shenzhen, Guangdong, China

## Abstract

Atopic dermatitis (AD) is a chronic inflammatory skin disease which is often associated with *Staphylococcus aureus* (*S. aureus*) colonization. *S. aureus* ingredients are potential ligands to activate the Toll-like receptor 2 (TLR2) and drive inflammatory cytokine or chemokine production. However, the role of TLR2-mediated chemokine expression in AD development has not been systematically investigated. In this study, we sought to determine the mode of TLR2-mediated chemokine expression in AD patients. Human peripheral blood mononuclear cells (PBMCs) were isolated from AD patients and healthy controls. Upon incubation with TLR2 ligands Pam3CSK4 and PGN, mRNA expression of chemokines, including CCL1, CCL5, CCL8, CCL13, CCL17, CCL18, CCL22, and CCL27, were determined by quantitative real-time polymerase chain reaction (qRT-PCR) analysis. The results showed that basal mRNA expression of CCL17 in PBMCs from AD patients was upregulated compared with healthy controls, while those of CCL8 and CCL13 were downregulated. When stimulated with TLR2 ligands, the mRNA expression of CCL5, CCL8, CCL13, CCL18, and CCL22 in PBMCs from AD patients was significantly higher than those from healthy controls. The different basal chemokine mRNA expression profiles indicate the different immune status in patients with AD compared with healthy controls. Excessive chemokine mRNA expression induced by TLR2 activation is associated with the development of AD.

## 1. Introduction

Atopic dermatitis (AD), which afflicts 10–20% of children and 1–3% of adults worldwide [1], is a chronic inflammatory disease characterized by eczematous skin lesion. Common symptoms include pruritus, relapsing eczema, and persistent cutaneous infection [2, 3]. Such symptoms usually occur in childhood and persist or vanish in adulthood. Genetic predisposition, allergen exposure, and epidermal barrier defect are generally believed to be involved in the progression of AD [2, 3]. Recent studies focus on the role of *Staphylococcus aureus* (*S. aureus*) infection and immune dysfunction in AD development [4, 5]. *S. aureus* colonization is associated with severe disease phenotype, T helper 2- (Th2-) skewing response and serious epidermal disruption in AD patients [5].

Toll-like receptor 2 (TLR2), one of the most important components of the pattern recognition receptors (PRRs), is widely expressed on cell membranes of immune cells and skin-resident cells, such as monocyte, eosinophil, T cell, dendrite cell (DC), and Langerhans cell (LC). TLR2 forms a heterodimer with TLR1 or TLR6 to recognize an extensive range of ligands. In general, TLR2/1 and TLR2/6 bind to various bacterial products, such as triacylated lipopeptides and diacylated lipopeptides, respectively. Tripalmitoyl-S-glycero-Cys-(Lys)4 (Pam3SCK4) is a synthetic triacylated lipopeptide which activates TLR2/1 heterodimer [6], while peptidoglycan (PGN) is a primary cell wall component of Gram-positive bacteria, such as *S. aureus*, which activates TLR2/6 heterodimer [7].

TLR2 is responsible for recognizing *S. aureus* products and initialing innate immunity. The colonization of *S. aureus* in lesional skins of AD patients resulted in enhanced expression of Th2 cytokines, such as interleukin (IL)-4, IL-13, and thymic stromal lymphopoietin (TSLP) [8]. When incubated with *S. aureus*-derived PGN, TLR2 induced CCL5, CCL17, and CCL22 production in LCs from AD patients [9, 10], suggesting that TLR2-mediated chemokine production may be a crucial process that protects against skin bacterial infection and contribute to the development of AD. Chemokine, a subclass of small cytokine proteins, regulates the immune response by migrating and localizing leukocytes. Enhanced levels of chemokines in AD skins are associated with the infiltration of immune cells, such as eosinophil, mast cell, monocyte, LC, and T cell [11]. Furthermore, increased serum levels of chemokines are closely related to the disease severity of AD [12].

In this study, we investigated the specific relationship between TLR2-chemokine signaling and AD progression. Peripheral blood mononuclear cells (PBMCs) were isolated from AD patients and healthy controls and incubated with TLR2 ligands Pam3SCK4 and PGN. Subsequently, the mRNA expression of chemokines, including CCL1, CCL5, CCL8, CCL13, CCL17, CCL18, CCL22, and CCL27, was determined by the qRT-PCR method.

## 2. Materials and Methods

### 2.1. Peripheral Blood Preparation

Peripheral blood samples were obtained from 40 patients suffering from moderate to severe AD, as designated by Hanifin and Rajka's criteria [13]. Of these 40 AD patients, 27 were female and 13 were male (mean age 31 years, with ages ranging from 20 to 45 years). Peripheral blood samples were also taken from 43 healthy controls (29 females and 14 males, with a mean age of 29 years and ages ranging from 20 to 42 years).

### 2.2. Isolation and Stimulation of PBMCs

PBMCs were isolated by standard Ficoll–Hypaque density gradient centrifugation from both AD patients and healthy controls. The cells were washed with PBS buffer and maintained at a density of 1x10^6^/mL in a RPMI 164 medium, supplemented with 2 mM L-glutamine, 100 U/mL penicillin-streptomycin, 10% fetal bovine serum (all purchased from Invitrogen, Carlsbad, USA), and 55 *μ*M 2-Mercaptoethanol (GIBCO, Eggestein, Germany). PBMCs were then stimulated with 1 *μ*g/mL Pam3CSK4 (Invivogen, San Diego, California, USA) or PGN (Sigma-Aldrich, Deisenhofen, Germany) for 8 hours. A preliminary experiment on apparent mRNA expression was conducted to select an optimal time point.

### 2.3. RNA Extraction and qRT-PCR Analysis

After 8 hours of stimulation with TLR2 ligands, total RNAs were extracted for quantification. mRNAs were collected by TRIzol reagents, then quantified by monitoring the ratio of spectrophotometric absorbance at 260 nm and 280 nm. mRNAs (1 *μ*g) were reverse transcribed into cDNAs using a Revert Aid™ First Strand cDNA Synthesis Kit (Thermo scientific, Waltham, MA, USA). The reverse transcribed mixtures were incubated for 50 min at 42°C, followed with inactivation of the enzyme by heating for 15 min at 70°C. cDNAs were amplified by target-specific primers (100 nM) and SYBR Green Supermix Kit (Bio-Rad, Hercules, CA, USA). The protocol contained an initial cycle (95°C for 5 min) followed by 40 amplifying cycles (denaturing at 95°C for 15 s, annealing at 60°C for 30 s, and then polymerizing at 72°C for 30 s). The melting curves were analyzed to identify PCR products. The 2^-*ΔΔ*Ct^ method was then applied to figure out the expression of target genes (chemokines) in ligand-stimulated cells relative to unstimulated ones, which normalized to the internal control (GAPDH). The CT values were calculated as follows:

(1) $$\begin{array}{c} \Delta \text{CT}={\text{CT}}_{\text{chemokine}}-{\text{CT}}_{\text{GAPDH}} \end{array}$$

(2) $$\begin{array}{c} \Delta \Delta \text{CT}={\text{CT}}_{\text{ligand}-\text{stimulated patient or control}}-{\text{CT}}_{\text{unstimulated ones}} \end{array}$$

The results were performed as fold changes of chemokine expression in AD patients and healthy controls.

### 2.4. Statistical Analysis

All experiments were conducted in triplicates. The Mann-Whitney *U* test was used for the comparison between AD patients and healthy controls. *p* < 0.05 was considered a significant difference.

## 3. Results

### 3.1. Increased CCL17, but Decreased CCL8 and CCL13 mRNA Expression in PBMCs from AD Patients

**Figure 1:**
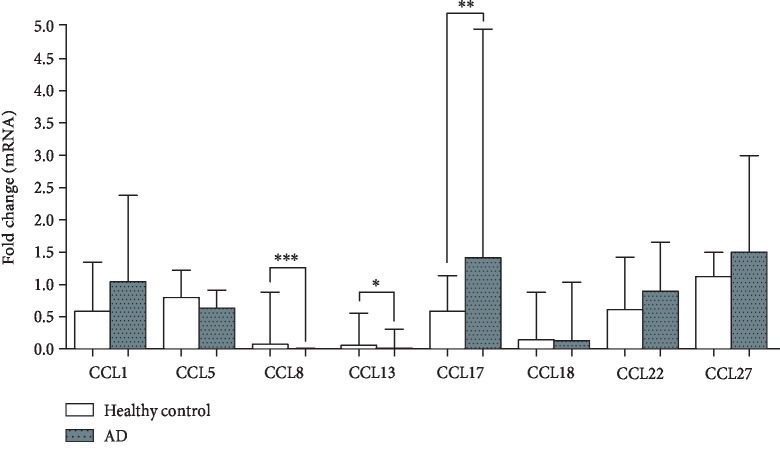
Basal levels of chemokines mRNA in PBMCs from AD patients and healthy controls. The mRNA expression of CCL1, CCL5, CCL8, CCL13, CCL17, CCL18, CCL22, and CCL27 were determined by qRT-PCR. Data are presented as median and interquartile range. Mann-Whitney *U* test was applied to compare the significant difference between the two groups. ^∗^*p* < 0.05, ^∗∗^*p* < 0.01, ^∗∗∗^*p* < 0.001.

We first determined the basal mRNA levels of chemokines in PBMCs from AD patients and healthy controls. As shown in the figure, PBMCs from AD patients showed significantly higher CCL17 but lower CCL8 and CCL13 mRNA expression than those from healthy controls (Figure 1).

### 3.2. Excessive mRNA Expression of CCL5, CCL8, CCL13, CCL18, and CCL22 in TLR2 Ligand-Stimulated PBMCs from AD Patients

**Figure 2:**
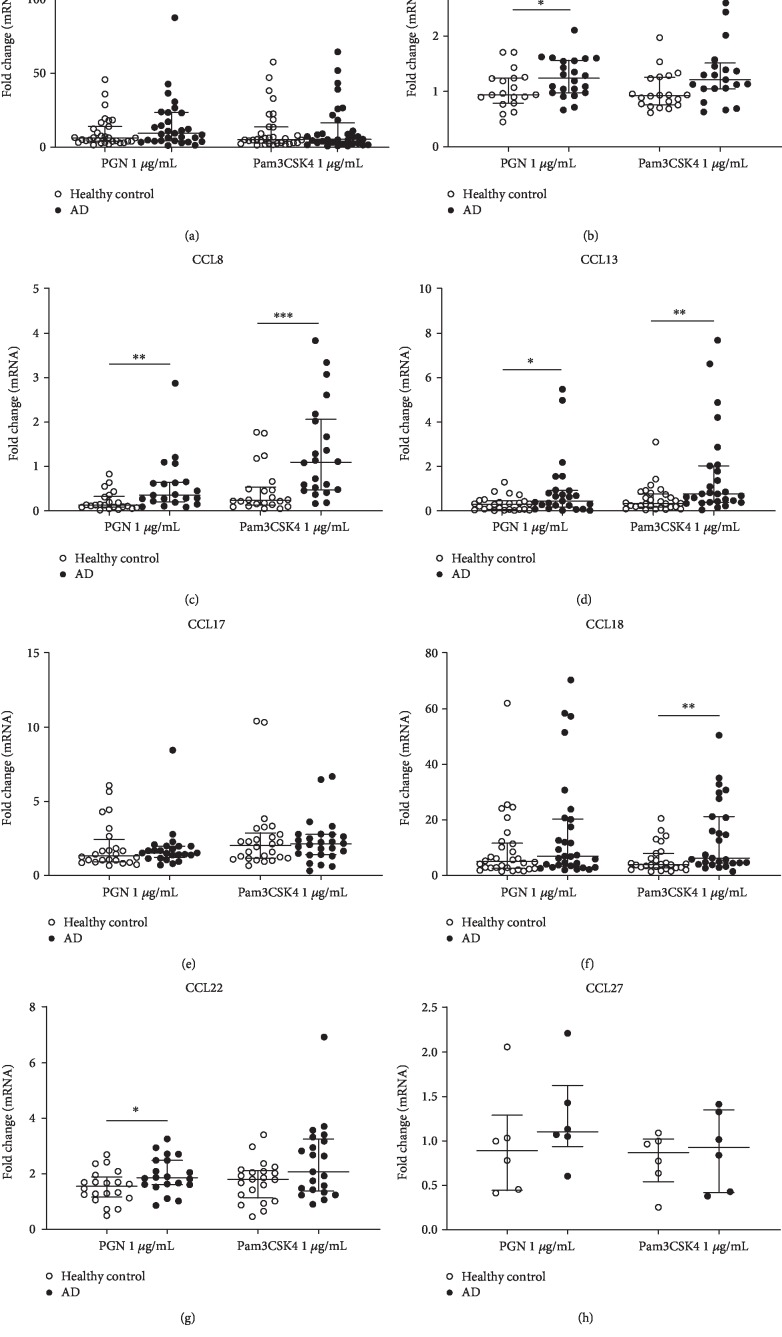
Effects of TLR2 ligands on chemokines mRNA expression in PBMCs from AD patients and healthy controls. After being incubated with TLR2 ligands Pam3SCK4 or PGN for 8 hours, the mRNA expression of CCL1 (a), CCL5 (b), CCL8 (c), CCL13 (d), CCL17 (e), CCL18 (f), CCL22 (g), and CCL27 (h) were determined by qRT-PCR. Data are presented as median and interquartile range. Mann-Whitney *U* test was applied to compare the significant difference between two groups. ^∗^*p* < 0.05, ^∗∗^*p* < 0.01, ^∗∗∗^*p* < 0.001.

We further determined whether TLR2/1 and TLR2/6 ligands are associated with the mRNA expression of chemokines in PBMCs from AD patients and healthy controls. PBMCs were stimulated with TLR2/6 ligand PGN (1 *μ*g/mL) or TLR2/1 ligand Pam3SCK4 (1 *μ*g/mL) for 8 hours. The mRNA expression of chemokines was measured by qRT-PCR. Upon TLR2 ligand stimulation, the mRNA expression of CCL5, CCL8, CCL13, CCL18, and CCL22 from AD groups were significantly higher than those from healthy controls (Figures 2(b)–2(d), 2(f), and 2(g)). On the other hand, both TLR2 ligands had no significant effect on the mRNA expression of CCL1, CCL17, and CCL27 in PBMCs from AD patients compared with healthy controls (Figures 2(a), 2(e), and 2(h)).

## 4. Discussion

AD is one of the most common types of chronic inflammatory skin disease. The debut of AD in infancy and subsequent occurrence of other allergy-associated diseases such as food allergy, asthma, and allergic rhinitis in childhood are known as the atopic march [2]. The inflammation of AD is known to be a biphasic reaction. The acute phase is mediated by Th2-type allergic response predominately with the production of IL-4, IL-5, IL-13, and TSLP, while the chronic phase is regulated by both Th2-response and Th1-type inflammatory response, with the production of IFN-*γ* [11].

AD patients are prevalence at TLR2 polymorphisms, and these mutant phenotypes are susceptible to *S. aureus* infection and perform severe disease activity [14, 15]. TLR2 ligands induced the secretion of chemokines CCL20, CCL2, and IL-8 in keratinocytes from AD patients, indicating that TLR2-chemokine signaling might involve in AD development [16]. On the one hand, the production of Th1 cytokines IFN-*γ* in PBMCs was suppressed by TLR2 ligands in AD subjects [17]. On the other hand, TLR2 ligands upregulated the expression of Fc*ε*RI, a high-affinity receptor for IgE, on PBMCs surface from AD patients [18]. *S. aureus*-derived TLR2 ligands promoted Th2-type response by enhancing TSLP production in keratinocytes [19]. Our study also demonstrated that TLR2 activation promoted PBMCs from AD patients expressed higher chemokines CCL5, CCL8, CCL13, CCL18, and CCL22, which mainly recruited eosinophils and Th2 cells. These phenomena indicate that TLR2 is likely to turn the Th1/Th2 balance towards Th2 deviation and exacerbate AD inflammation.

Many chemokines are associated with allergic diseases, such as AD, asthma, and arthritis [20, 21]. They are implicated in the progression of AD by recruiting immune cells into lesional skin [22]. In this study, increased CCL17 and decreased CCL8 and CCL13 mRNA expression were observed in PBMCs from AD patients. The promotion of Th2 chemokines CCL17 mRNA aggravate clinical symptoms in AD patients. However, the suppression of CCL8 and CCL13 mRNA may attribute to selectively suppression of excessive inflammatory response. These findings indicate that there is a different expression profile of chemokines between AD patients and healthy controls. Furthermore, with the activation by TLR2 ligands, AD patients showed significantly higher mRNA expression of CCL5, CCL8, CCL13, CCL18, and CCL22 in PBMCs. These results indicate that TLR2 plays a critical role in promoting chemokine expression and results in excessive inflammatory response in AD progression. Due to the lack of enough blood samples, further studies should elucidate the effect of TLR2 activation on chemokine release of PBMCs from AD patients by ELISA, and phenotypic characterization of PBMCs could be done to better investigate the mechanism of AD.

Interaction of CCL5, CCL8, and CCL13 with CC chemokine receptors (CCR) 3 are responsible for the recruitment of eosinophils [23]. Previous studies have shown that AD increased the production of CCL5 in both lesional skins and peripheral blood [24, 25]. TLR2/6 ligand PGN promoted epidermal LCs to release CCL5 in a p38-MAPK-dependent manner and subsequently recruited eosinophils into the inflamed skin [10, 22]. CCL8 induced eosinophilia through recruiting IL-5-producing CCR8+Th2 cell in AD murine model [26]. Meanwhile, the CCL8-CCR8 axis is important in promoting the migration of skin DCs to draining lymph nodes and triggering Th2 deviation for allergic inflammation [27]. In addition, CCL13 and CCL20 recruited circulating DC precursors from vessel to inflamed skin in a sequential manner [28].

CCL17, CCL22, and CCL18, ligands of CCR4 and CCR8, respectively, served as chemoattractants for recruiting Th2 cell [23]. Enhanced secretion of CCL17 and CCL22 was observed in epidermal LCs from AD patients upon TLR2/6 ligand PGN incubation [9]. CCL17 and CCL22 were associated with the Th2-type response of AD and could serve as favorable biomarkers for predicting disease severity [12, 29]. Applied appropriate treatments for AD showed lower serum CCL17 level [30], while knockdown of CCL22 gene reduced IL-4 and IgE but induced IFN-*γ* production [29]. In addition, enhanced expression of CCL22 was associated with the maturation course of LCs from sensitized skins to lymph node [31]. CCL18 is the most highly expressed chemokines by antigen-presenting cells (APCs) in AD and asthma subjects. [20]. CCL18-expressing cells are accumulated in lesional skin [32], and the level of serum CCL18 concentration was also associated with the AD severity, serum eosinophil, and IgE levels [21]. Upon incubation with Th2 cytokines IL-4 and IL-13, there were higher proportions of CCL18-producing cells present in PBMCs from AD patients [33].

Epidermal barrier defect facilitates *S. aureus*-derived products to penetrate to the epidermis, later influx in peripheral vessel. TLR2 is responsible for recognizing these products and initialing innate immunity. TLR2 enhanced chemokine expression in the primary immune cell, such as PBMCs component macrophages and DC precursors, representing a systemic defense against bacterial infection. These chemokines will bind to corresponding receptors and attract more circulating immune cells into inflamed skin [22, 24, 26, 34]. We have summarized the receptors and key functions of these chemokines in the context of AD milieus in Table 1, partly refers to two articles [35, 36]. In general, upon TLR2 activation, CCL5, CCL8, and CCL13 work together in recruiting eosinophil, while CCL17, CCL22, and CCL18 are attracting Th2 cells into the skin. These immune cells create a Th2-dominant milieu with the recruitment of eosinophils and Th2 cells in lesional skin, exacerbate cutaneous allergic-like inflammation during persistent pathogen exposure. In addition, circulating DC precursors are mobilized across the vessel and emigrate into inflamed skins to recognize antigens with the assistance of CCL13. Subsequently, CCL8 and CCL22 guide APCs (DC precursors or LCs) to draining lymphatic tissues, during the DC maturation process. That is, TLR2-chemokine signaling contribute to bridge the crosstalk between innate and adaptive immunity.

Suppression of TLR2-chemokine signaling are effective in controlling the acute inflammatory response of AD patients. For example, CCR4 antagonist or modified CCL5 peptide reduced T cell and eosinophil infiltration and also attenuated AD-like skin injury [37, 38]. In addition, Dupilumab, a monoclonal antibody against IL-4 receptor *α*, significantly suppressed the expression of Th2-type chemokines (CCL17, CCL18, CCL22, and CCL26) and had been approved by FDA for the therapy of moderate-to-severe AD in adult patients [39]. Considering that the excessive chemokines expression upon TLR2 activation in AD patients, the anti-chemokine therapies or blockage of upstream TLR2 signaling would be a new alternative treatment for AD.

## Figures and Tables

**Table 1:** The receptors and function properties of chemokines in atopic dermatitis.

Chemokines	Another names	Original cells	Receptors	Target cells	Key functions
CCL5	RANTES	Eosinophil, keratinocyte, LC	CCR1, CCR3, CCR5	Eosinophil, fibroblast, T cell	Eosinophil migration
CCL8	MCP-2	Fibroblast, monocyte	CCR1, CCR2, CCR3, CCR5 (human), CCR8 (mouse)	Basophil, eosinophil, DC precursor, monocyte, Th2 cell	Th2 response, eosinophil migration
CCL13	MCP-4	Endothelial cell, epithelial cell, keratinocyte, monocyte, T cell	CCR2, CCR3, CCR5	Eosinophil, DC precursor, monocyte, T cell	Th2 response, eosinophil migration
CCL17	TARC	DC, endothelial cell, fibroblast, keratinocyte	CCR4, CCR8	Th2 cell, Treg cell	Th2 response, Th2 cell migration
CCL18	PARC, DC-CK1	DC/LC, keratinocyte, monocyte	CCR8	DC precursor, T cell	Th2 response, skin homing of T cell
CCL22	MDC	DC, monocyte	CCR4	Th2 cell, Treg cell	Th2 response, Th2 cell and DC migration

## Data Availability

The data used to support the findings of this study are available from the first author upon reasonable request.
